# Estimates of walking prevalence and volume for U.S. cancer survivors and those without cancer: overall, by sex, and by race and ethnicity

**DOI:** 10.1007/s11764-024-01729-6

**Published:** 2024-12-19

**Authors:** Calvin P. Tribby, Sarah Alismail, Nivedita Nukavarapu, Jiue-An Yang, Marta M. Jankowska

**Affiliations:** 1 Department of Population Sciences, Lippman Graff Building, Beckman Research Institute, City of Hope, Office #B123, 1500 E Duarte Rd, Duarte, CA 91010 USA; 2 Windreich Department of Artificial Intelligence and Human Health, Icahn School of Medicine at Mount Sinai, Sinai, NYC USA

**Keywords:** Exercise, Malignant neoplasms, Recreation, Purposeful walking, Active travel, Utilitarian walking

## Abstract

**Purpose:**

This paper estimated overall, by sex, and by race and ethnicity walking behaviors in the cancer survivor population, where prevalence is not known, compared to those without cancer.

**Methods:**

Data from the 2015 and 2020 National Health Interview Survey (*n* = 54,542) were used to estimate walking behaviors. Multivariable logistic regression models estimated walking behavior prevalence with predictive margins and volume of weekly minutes overall and stratified by sex and race/ethnicity. Walking behaviors for breast and prostate cancer survivors were also examined.

**Results:**

There were no significant differences in adjusted prevalence for walking behaviors overall or by race/ethnicity for women. However, there were significant differences for men, with cancer survivors’ any reported walking at 62.6% (95% CI: 60.1, 65.4) compared to men without cancer, 65.9% (95% CI: 65.1%, 66.8%) (*p* = 0.02). There was also a difference in transportation only walking for men, with cancer survivors reporting 6.8% (95% CI: 5.5%, 8.2%), compared to men without cancer, 9.1% (8.5%, 9.6%) (*p* = 0.008); a similar pattern was observed for transportation walking for non-Hispanic white men. There were no differences in walking prevalence among breast cancer survivors, but overall prostate cancer survivors reported less walking for both purposes as did non-Hispanic white survivors. Leisure walking volume for cancer survivors, both women and men, was higher than for those without cancer. Median leisure walking minutes for non-Hispanic white women, 120 min (95% CI: 120, 140) was higher than those without cancer, 105 min (95% CI: 105, 120) (*p* = 0.002). Median leisure walking minutes for non-Hispanic white men, 120 min (95% CI: 120, 140), was higher than those without cancer, 100 min (95% CI: 100, 105) (*p* = 0.001).

**Conclusions:**

Overall, there are no significant differences in walking prevalence for women, but men cancer survivors reported less overall walking, walking for transportation, or walking for both purposes. However, volume of leisure walking was higher for cancer survivors compared to those without cancer.

**Implications for Cancer Survivors:**

For cancer survivors, this suggests that even though prevalence of leisure walking was similar, volume of weekly minutes was higher compared to those without cancer. This suggests that for cancer survivors, leisure walking is an accessible and important source of physical activity.

**Supplementary information:**

The online version contains supplementary material available at 10.1007/s11764-024-01729-6.

## Background

The number of cancer survivors in the USA was estimated to be 18 million individuals in 2022 and is projected to grow to over 22.1 million in 2030 [1, 2]. Research has examined a wide range of health behaviors of cancer survivors, such as diet, physical activity, tobacco or alcohol use, sleep, sun protection, and vaccination uptake [3–8]. Racial and ethnic differences in health behaviors of cancer survivors are not investigated as thoroughly. Some research indicates that behaviors such as diet, physical activity, and alcohol use varied among cancer survivor racial/ethnic groups [9–11]. For example, non-Hispanic Black and Hispanic cancer survivors had higher adherence to alcohol guidelines (at that time: 2 or fewer drinks per day for men; 1 or fewer drinks per day for women) compared to non-Hispanic whites, but there was no significant difference in physical activity among the three racial/ethnic groups [9].

Ongoing surveillance of aerobic physical activity (PA) in both cancer survivors and those without cancer is important for the evaluation of PA guideline adherence, Healthy People 2030 benchmarks, and identification of correlates for potential differences between the two populations [12–15]. Additionally, PA has been identified as an important behavioral intervention for improving health of cancer survivors as well as reducing cancer risk in the general population [16, 17]. Most research has focused on leisure-time moderate to vigorous PA (MVPA), yet emerging evidence suggests health benefits of light intensity PA, such as lower all-cause mortality [18–21]. While there are still some research areas that need more evidence regarding the potential benefits of light intensity PA [22], there is evidence that this lower intensity PA has health benefits for cancer survivors [23] and may be more flexible and achievable for those with cancer-related fatigue or other adverse treatment-related symptoms [24, 25].

Walking is an important component of PA, being the most common type of PA overall [26] and among cancer survivors [27], requires no special equipment, has very little associated costs, and walking is a broadly accessible activity to start or maintain physical activity for the general population and groups that are more sedentary [12, 28]. There are two main purposes of walking: for leisure or transportation [12, 29]. These purposes are important to disaggregate as there are sex and socioeconomic differences in their prevalence [12, 29–31]. While general PA variations between cancer survivors and those without cancer have been examined [5, 7, 10, 32, 33], walking behavior explicitly has not yet been examined. This is a gap in understanding PA behaviors of these two populations, as walking is an important component of PA, yet is not entirely captured by survey questions that solely ask about leisure-time PA. Walking for transportation (not for leisure, but to get some place, such as a transit stop, work, or a store) is not likely captured by leisure-time PA questions in national health surveys, where participants may only report their leisure-time walking activity [34].

Racial and ethnic differences in walking behaviors are not well understood, with most research focusing on perceptions or physical barriers of walking environments [35–37]. Furthermore, whether there are racial and ethnic differences in walking behaviors of cancer survivors compared to those without cancer remains largely unknown. Identifying possible walking behavior differences may help to broaden understanding of larger population-level cancer and survivorship disparities. This study expands on previous analyses into racial and ethnic differences in general health behaviors among cancer survivors [9, 10]. The goals of this study are to examine the potential racial and ethnic differences in walking between cancer survivors and those without cancer by (1) estimating the prevalence of walking, both overall and based on walking purpose; (2) estimating the prevalence of walking for the most common cancer in men (prostate) and women (breast) to account for potential variations in physical activity levels due to cancer site [7]; and (3) estimating the volume of weekly minutes of walking among these populations.

## Methods

### Study sample

This research used nationally representative 2015 and 2020 National Health Interview Surveys (NHIS; 2015 *N* = 33,672; 2020 *N* = 31,568). The NHIS is an annual household survey of the U.S. noninstitutional adult (age ≥ 18 years) population [38]. These 2 years correspond to when specific questions on walking were fielded in the Cancer Control supplement module [39]. We pooled these years to address small numbers in the cancer survivor population to examine walking behaviors in racial and ethnic groups. As this was the primary focus of this study and the NHIS does not include questions on cancer stage at diagnosis or treatment, we combined all cancer types and time since diagnosis to examine overall differences in walking prevalence and separately for breast and prostate cancers. This study was exempt from review by the City of Hope institutional review board because it used publicly available deidentified data.

We excluded respondents who were unable to walk (*n* = 1418) or had missing responses for any variables (*n* = 9280). We examined time since cancer diagnosis and found that it was missing for some respondents (*n* = 62). However, considering the small number of cancer survivors, we coded it as unknown/missing and did not exclude these participants from the analysis. The final analytic sample was *n* = 54,542.

### Measures

We defined cancer survivors as those who reported ever being told by a doctor or health professional that they had cancer or a malignancy of any kind. Participants were defined as cancer survivors starting from their initial diagnosis through the year of survey [40, 41]. We utilized self-report of any cancer to define the overall cancer survivor population and then examined the most common incident cancers by sex (men: prostate; women: breast) [42].

To determine walking for transportation, respondents were asked in 2015: “During the past 7 days, did you walk to get someplace that took you at least 10 min?” To assess leisure-time walking, respondents were asked in 2015: “During the past 7 days, did you walk for at least 10 min at a time [for fun, relaxation, exercise, or to walk the dog]? Please do not include walking for transportation.” The 10-min bout limitation was removed for the 2020 survey questions, which aligns with the updated PA guidelines [13]. These questions were used to group respondents into categories to determine prevalence of different walking behaviors: any walking (yes to either question); walking only for transportation (yes to transportation and no to leisure questions); walking only for leisure (no to transportation and yes to leisure questions); and walking for both categories (yes to transportation and yes to leisure questions) [29, 34]. Respondents who answered yes to either walking question were asked: “In the past 7 days, how many times did you do that [walk for leisure or transportation]?” and “On average, how long did those walks take?” For each participant, the volume of walking (minutes per week) was estimated by multiplying the number of bouts per week by the average minutes per bout.

Demographics and measures related to health status or walking were age category (18–49; 50–64; or 65 +), leisure-time PA (inactive, insufficiently active, sufficiently active, or highly active) [13], weight status (under/normal, overweight, or obese), difficulty walking (any or none) [26], health insurance status (any or none), education level (high school or less, some or college degree, graduate or professional degree), marital status (married or living with partner, divorced/separated, or never married), employment status (yes or no), any functional limitations (any or none) [6], time since cancer diagnosis (unknown/missing, early (< 3 years), short (3–5 years), long (≥ 5 years)) [6], and survey year.

### Statistical analyses

We examined differences in any walking and mutually exclusive walking purpose categories (transportation only; leisure only; both transportation and leisure walking) by cancer survivor status. We included any reported cancers in the cancer survivor group because a sensitivity analysis did not find a significant difference in prevalence of any reported walking, when comparing estimates excluding non-melanoma skin cancer survivors and including them (difference in proportions *p* = 0.10). We assessed unadjusted and adjusted walking prevalence stratified by sex [12] and race/ethnicity (Hispanic ethnicity, any race; non-Hispanic white; non-Hispanic Black; and non-Hispanic other or multiple). We tested the interaction between race/ethnicity and cancer status on any walking and found a significant interaction (*p* < 0.0001), so we further stratified by race/ethnicity. We examined differences in walking volume by estimating subgroup medians, as minutes of weekly walking is right skewed [34]. Overall adjusted models control for all covariates. Models stratified by sex do not control for sex. Similarly, models stratified by sex and race/ethnicity do not control for these two variables.

The data were analyzed with R, version 4.2.2 (R Foundation for Statistical Computing, Vienna, Austria), the *survey* package, version 4.1–1 (*svydesign*, *svyglm*, *svyranktest*, and *svyquantile* functions) [43], and the *srvyr* package, version 1.2.0 (*survey_prop*, *svypredmeans* and *svychisq* functions) [44]. These packages account for the complex sampling design, weights, and perform subgroup analyses. For unadjusted proportions, we used the survey-based Clopper-Pearson confidence interval estimates for small prevalence correction [45]. As suggested by the National Center for Health Statistics, the sample weights were divided by 2 because there were 2 years of data [46]; this sample weight adjustment ensures that sample respondents were not overweighted. Also, because the 2 sample years were within different sample design periods (design periods are updated based on the decennial census; the NHIS was re-designed in 2019), the data from the 2 years were treated as statistically independent samples, and the stratum variables were updated to reflect the different sample design periods [46].

Walking prevalence was estimated with predictive margins from multivariable logistic regression models and significance tests for categorical variables used the adjusted Wald F-test. Covariates in adjusted analyses were age category, leisure-time PA, weight status, difficulty walking, health insurance status, education level, marital status, employment status, any functional limitations, time since cancer diagnosis, and survey year.

For participants who reported any walking, significance tests for median walking minute differences used Mood’s test for the median and median confidence intervals reported the “xlogit” interval calculation method to match SUDAAN estimates [47]; *p* < 0.05 is the level of significance, and 95% confidence intervals are presented with all point estimates. Analyses were conducted in 2023.

## Results

### Sample description

Overall, 7.8% (95% CI: 7.5%, 8.1%) of the U.S. population are estimated to be cancer survivors from this sample. Table 1 presents descriptive statistics for the cancer survivors and those without cancer groups. The overall unadjusted prevalence of any walking for cancer survivors was 63.8% (62.1%, 65.6%), and for those without cancer was 67.3% (66.6%, 67.9%) (Table 1). The prevalence of walking for transportation was lower for cancer survivors, 5.6% (4.8%, 6.5%), compared to those without cancer, 8.3% (7.9%, 8.6%). Finally, walking for both purposes was lower for cancer survivors, 12.4% (11.2%, 13.7%), compared to those without cancer, 16.6% (16.1%, 17.1%). Prevalence of any functional limitations was higher for cancer survivors, 33.9% (95% CI: 32.1%, 35.8%), compared to those without cancer, 17.2% (95% CI: 16.7%, 17.4%) (Table 1). Also, prevalence of any difficulty walking was higher for cancer survivors, 10.7% (95% CI: 9.7, 11.8%), compared to those without cancer, 3.8% (95% CI: 3.6%, 4.0%) (Table 1).

**Table 1:** Sample description of variables stratified by cancer survivor status, NHIS 2015 and 2020

	Cancer survivors *n* = 5670		Those without cancer *n* = 48,872	
	Unweighted (*n*)	Weighted % (95% CI)	Unweighted (*n*)	Weighted % (95% CI)
Sex				
Female	3166	54.1 (52.4, 55.7)	25,814	50.1 (49.5, 50.7)
Male	2504	45.9 (44.3, 47.6)	23,058	49.9 (49.3, 50.5)
Age				
18–49	682	15.6 (14.3, 17.1)	25,942	61.0 (60.4, 61.6)
50–64	1702	32.9 (31.2, 34.6)	13,493	25.7 (25.1, 26.2)
65 +	3286	51.5 (49.7, 53.2)	9437	13.3 (13.0, 13.7)
Race/ethnicity				
Hispanic, any race	310	6.0 (5.1, 7.1)	7706	17.3 (16.5, 18.2)
White, non-Hispanic	4828	85.0 (83.6, 86.3)	31,166	62.2 (61.3, 63.2)
Black, non-Hispanic	346	5.7 (5.0, 6.6)	5691	11.6 (11.0, 12.2)
Other, non-Hispanic	186	3.3 (2.7, 4.0)	4309	8.8 (8.3, 9.4)
Weight status				
Under/normal	1965	33.6 (31.9, 35.3)	16,918	35.1 (34.5, 35.7)
Overweight	2075	36.8 (35.3, 38.4)	16,950	34.0 (33.4, 34.5)
Obese	1630	29.6 (28.0, 31.1)	15,004	31.0 (30.4, 31.5)
Time since diagnosis				
Unknown/missing	62	1.2 (0.8, 1.7)	–	n/a
Early (< 3 years)	1279	22.6 (21.1, 24.2)	–	n/a
Short (3–5 years)	683	12.5 (11.4, 13.7)	–	n/a
Long (≥ 5 years)	3646	63.6 (61.9, 65.4)	–	n/a
Physical activity level				
Inactive	1662	29.2 (27.5, 30.9)	12,641	25.5 (24.9, 26.2)
Insufficiently active	1372	24.9 (23.5, 26.5)	11,295	23.5 (23.0, 24.1)
Sufficiently active	1004	17.2 (16.0, 18.5)	8968	18.1 (17.6, 18.5)
Highly active	1632	28.7 (27.1, 30.3)	15,968	32.8 (32.2, 33.5)
Functional limitations				
Any	1925	33.9 (32.1, 35.8)	9835	17.2 (16.7, 17.4)
None	3745	66.1 (64.2, 67.9)	39,037	82.8 (82.3, 83.3)
Difficulty walking				
Any	639	10.7 (9.7, 11.8)	2372	3.8 (3.6, 4.0)
None	5031	89.3 (88.2, 90.3)	46,500	96.2 (96.0, 96.4)
Any walking	3667	63.8 (62.1, 65.6)	33,169	67.3 (66.6, 67.9)
No walking	2003	36.2 (34.4, 37.9)	15,703	32.7 (32.1, 33.4)
Transportation walking only	319	5.6 (4.8, 6.5)	4245	8.3 (7.9, 8.6)
Leisure walking only	2617	45.8 (44.0, 47.6)	20,498	42.4 (41.7, 43.1)
Both walking types	731	12.4 (11.2, 13.7)	8426	16.6 (16.1, 17.1)
Health insurance status				
Any	5513	96.3 (95.4,97.0)	43,807	88.3 (87.7, 88.7)
None	157	3.7 (3.0, 4.6)	5065	11.7 (11.3, 12.3)
Marital status				
Married or living with partner	3139	68.6 (67.2, 70.0)	26,211	60.9 (60.3, 61.6)
Divorce/separated	2029	24.8 (23.5, 26.1)	11,111	14.2 (13.8, 14.6)
Never married	502	6.6 (5.9, 7.4)	11,550	24.9 (24.3, 25.5)
Employment status				
Employed	1983	38.2 (36.5, 39.9)	31,653	67.6 (67.0, 68.2)
Not employed	3687	61.8 (60.1, 63.5)	17,219	32.4 (31.8, 33.0)
Survey year				
2015	2691	49.5 (47.6, 51.4)	26,000	49.5 (48.5, 50.5)
2020	2979	50.5 (48.6, 52.4)	22,872	50.5 (49.5, 51.5)

### Adjusted walking prevalence

For brevity, unadjusted results for comparison to the following adjusted analyses are presented in the online supplement, but not discussed here. The overall covariate-adjusted prevalence of any walking for cancer survivors was 65.7% (95% CI: 64.0%, 67.5%), and for those without cancer, it was 67.1% (95% CI: 66.5%, 67.7%). For women, the adjusted prevalence of any reported walking for cancer survivors was 68.0% (95% CI: 65.8%, 70.1%), and for those without cancer, it was 68.2% (95% CI: 67.4%, 69.0%) (*p* = 0.80) (Table 2). There were no significant racial/ethnic differences in the prevalence of walking behaviors between women cancer survivors and those without cancer (Table 2). For men, the adjusted prevalence of any walking for cancer survivors was 62.8% (95% CI: 60.1%, 65.4%) and for those without cancer was 65.9% (95% CI: 65.1%, 66.8%) (*p* = 0.02) (Table 2). Men cancer survivors reported a lower prevalence of walking for transportation only: 6.8% (95% CI: 5.5%, 8.2%) compared to those without cancer with 9.1% (95% CI: 8.5%, 9.6%) (*p* = 0.008) (Table 2). Also, men cancer survivors reported a lower prevalence of walking for both purposes: 14.7% (95% CI: 12.6%, 16.9%) compared to those without cancer 17.6% (95% CI: 16.9%, 18.3%) (*p* = 0.02). One significant racial/ethnic difference in walking prevalence was observed for non-Hispanic white men, where cancer survivors had lower any walking prevalence, 64.6% (95% CI: 61.8%, 67.3%), compared to those without cancer, 67.6% (95% CI: 66.6%, 68.7%) (*p* = 0.03) (Table 2). Also, non-Hispanic white men cancer survivors had lower transportation walking prevalence, 6.4% (95% CI: 4.9%, 7.9%), compared to those without cancer, 8.6% (95% CI: 8.0%, 9.3%) (*p* = 0.02) (Table 2).

**Table 2:** Adjusted predicted walking prevalence (% and 95% CI) comparing all cancer survivors to those without cancer for women and men overall and stratified by race/ethnicity, NHIS 2015 and 2020

	Overall			Race/ethnicity											
				Hispanic			White, non-Hispanic			Black, non-Hispanic			Other, non-Hispanic		
**Women**	Cancer survivors *n* = 3166	Those without cancer *n* = 25,814	*p*	Cancer survivors *n* = 197	Those without cancer *n* = 4133	*p*	Cancer survivors *n* = 2,648	Those without cancer *n* = 16,080	*p*	Cancer survivors *n* = 193	Those without cancer *n* = 3366	*p*	Cancer survivors *n* = 128	Those without cancer *n* = 2235	*p*
Any reported walking	68.0 (65.8, 70.1)	68.2 (67.4, 69.0)	0.80	70.1 (61.9, 78.2)	66.0 (63.9, 68.0)	0.35	69.3 (67.0, 71.6)	69.7 (68.8, 70.7)	0.73	56.9 (48.0, 65.7)	59.9 (57.5, 62.2)	0.50	74.2 (64.6, 83.7)	73.1 (70.8, 75.4)	0.82
Transportation only	7.0 (5.4, 8.6)	7.2 (6.7, 7.6)	0.79	16.0 (6.0, 26.0)	9.4 (8.2, 10.5)	0.12	5.3 (3.9, 6.8)	5.7 (5.2, 6.2)	0.65	9.3 (4.2, 14.4)	10.5 (9.1, 12.0)	0.66	4.7 (0.1, 9.5)	9.3 (7.8, 10.7)	0.18
Leisure only	46.5 (44.0, 48.9)	45.9 (45.0, 46.8)	0.68	43.8 (34.9, 52.7)	41.8 (39.6, 43.9)	0.66	49.3 (46.7, 51.9)	49.2 (48.1, 50.3)	0.97	35.9 (26.8, 45.1)	34.9 (32.6, 37.2)	0.83	48.5 (37.2, 59.8)	44.8 (42.0, 47.6)	0.52
Both purposes	14.2 (12.4, 16.0)	15.1 (14.5, 15.8)	0.32	10.5 (5.7, 15.3)	14.9 (13.5, 16.2)	0.13	14.4 (12.3, 16.5)	14.8 (14.0, 15.6)	0.70	11.0 (5.1, 16.9)	14.4 (12.7, 16.0)	0.32	20.4 (10.6, 30.1)	19.0 (16.7, 21.3)	0.78
**Men**	*n* = 2504	*n* = 23,058		*n* = 113	*n* = 3573		*n* = 2,180	*n* = 15,086		*n* = 153	*n* = 2,325		*n* = 58	*n* = 2074	
Any reported walking	62.8 (60.1, 65.4)	65.9 (65.1, 66.8)	0.02	61.6 (48.4, 74.8)	60.5 (58.4, 62.6)	0.87	64.6 (61.8, 67.3)	67.6 (66.6, 68.7)	0.03	55.1 (44.6, 65.6)	60.6 (58.2, 63.1)	0.30	78.2 (63.9, 2.6)	69.6 (67.2, 72.1)	0.30
Transportation only	6.8 (5.5, 8.2)	9.1 (8.5, 9.6)	0.008	5.2 (0.8, 9.6)	8.9 (7.7, 10.0)	0.20	6.4 (4.9, 7.9)	8.6 (8.0, 9.3)	0.02	11.6 (6.0, 17.3)	11.2 (9.4, 13.0)	0.89	8.2 (0.02,16.3)	10.1 (8.6, 11.7)	0.67
Leisure only	40.7 (38.0, 43.3)	39.3 (38.4, 40.1)	0.31	43.2 (31.2, 55.1)	34.0 (32.0, 36.1)	0.12	42.9 (40.1, 45.7)	41.8 (40.7, 42.9)	0.45	26.7 (17.8, 35.6)	30.9 (28.3, 33.4)	0.39	48.3 (29.1, 67.5)	40.2 (37.5, 42.9)	0.40
Both purposes	14.7 (12.6, 16.9)	17.6 (16.9, 18.3)	0.02	13.8 (5.5, 22.1)	17.6 (15.9, 19.3)	0.41	14.7 (12.4, 17.1)	17.2 (16.4, 18.0)	0.06	17.4 (9.5, 25.4)	18.5 (16.4, 20.7)	0.79	21.7 (7.8, 35.6)	19.3 (17.0, 21.6)	0.73

*P*-values are from *t*-tests of any cancer variable coefficient in the models, not a comparison between predicted marginals. Covariates: leisure-time physical activity level, age, weight status, education, marital status, employment status, functional status, difficulty walking, health insurance status, and survey year. Overall model additionally includes race/ethnicity

### Breast or prostate cancer-specific walking prevalence

For women breast cancer survivors, there were no significant differences overall or by race/ethnicity for walking behaviors compared to those without cancer (Table 3). The one significant result for Hispanic women who reported transportation walking was potentially an unreliable estimate due to the small sample size (*n* = 18 cancer survivors) and large confidence interval (95% CI: 8.7%, 52.2%) (Table 3). Men prostate cancer survivors reported less walking for both purposes, 12.6% (95% CI: 9.3%, 15.9%), compared to those without cancer, 17.8% (95% CI: 17.1%, 18.5%) (*p* = 0.008) (Table 3). Non-Hispanic white prostate cancer survivors reported less walking for both purposes, 11.3% (95% CI: 7.9%, 14.8%), compared to those without cancer, 17.4% (95% CI: 16.6%, 18.3%) (*p* = 0.004) (Table 3).

**Table 3:** Adjusted predicted prevalence (% and 95% CI) of walking behavior for women breast cancer survivors and men prostate cancer survivors compared to those without cancer, overall and stratified by race/ethnicity, NHIS 2015 and 2020

	Overall			Race/ethnicity											
				Hispanic			White, non-Hispanic			Black, non-Hispanic			Other, non-Hispanic		
**Women**	Breast cancer survivors *n* = 1048	Those without cancer *n* = 25,814	*p*	Breast cancer survivors *n* = 75	Those without cancer *n* = 4133	*p*	Breast cancer survivors *n* = 836	Those without cancer *n* = 16,080	*p*	Breast cancer survivors *n* = 81	Those without cancer *n* = 3366	*p*	Breast cancer survivors *n* = 56	Those without cancer *n* = 2235	*p*
Any reported walking	67.2 (63.1, 71.3)	68.4 (67.7, 69.2)	0.57	67.7 (50.4, 85.0)	66.0 (63.9, 68.1)	0.85	68.6 (64.1, 73.0)	70.1 (69.2, 71.1)	0.49	57.3 (44.7, 70.0)	60.0 (57.7, 62.4)	0.68	73.9 (61.8, 86.0)	73.2 (70.9, 75.5)	0.91
Transportation only	9.3 (5.7, 12.9)	7.3 (6.8, 7.8)	0.23	30.4 (8.7, 52.2)	9.4 (8.2, 10.6)	0.008	7.2 (4.0, 10.4)	5.8 (5.2, 6.3)	0.34	5.0 (0.01, 10.9)	10.6 (9.1, 12.0)	0.20	3.6 (0.01, 9.4)	9.4 (7.9, 10.8)	0.23
Leisure only	44.1 (40.0, 48.2)	45.9 (45.0, 46.7)	0.39	28.7 (16.9, 40.5)	41.7 (39.5, 43.9)	0.05	47.4 (42.6, 52.1)	49.3 (48.2, 50.4)	0.41	36.4 (24.1, 48.8)	35.0 (32.7, 37.3)	0.82	51.1 (33.8, 68.6)	44.8 (42.0, 47.6)	0.47
Both purposes	13.9 (10.7, 17.1)	15.3 (14.6, 15.9)	0.41	10.9 (3.9, 17.8)	14.9 (13.5, 16.3)	0.34	13.8 (10.1, 17.6)	15.0 (14.2, 15.8)	0.55	14.7 (4.9, 24.4)	14.5 (12.8, 16.2)	0.97	17.7 (3.4, 32.0)	19.1 (16.8, 21.4)	0.87
**Men**	Prostate cancer survivors *n* = 737	Those without cancer *n* = 23,058	*p*	Prostate cancer survivors *n* = 42	Those without cancer *n* = 3573	*p*	Prostate cancer survivors *n* = 590	Those without cancer *n* = 15,086	*p*	Prostate cancer survivors *n* = 90	Those without cancer *n* = 2325	*p*	Prostate cancer survivors *n* = 15	Those without cancer *n* = 2074	*p*
Any reported walking	61.8 (56.9, 66.6)	66.0 (65.1, 66.8)	0.09	66.6 (49.3, 83.9)	60.4 (58.4, 62.6)	0.51	64.5 (59.5, 69.6)	67.8 (66.8, 68.9)	0.19	51.9 (38.0, 65.7)	60.8 (58.3, 63.3)	0.21	69.4 (35.2, 0.99)	69.6 (67.1, 72.0)	0.99
Transportation only	7.0 (4.0, 9.3)	9.1 (8.6, 9.7)	0.12	7.6 (0.01, 17.1)	8.8 (7.7, 10.0)	0.81	5.8 (2.8, 8.7)	8.7 (8.0, 9.4)	0.12	11.6 (4.4, 18.9)	11.3 (9.4, 13.1)	0.92	10.3 (0.01, 27.7)	10.2 (8.6, 11.7)	0.99
Leisure only	41.7 (36.8, 46.6)	39.1 (38.2, 39.9)	0.29	43.7 (24.2, 63.3)	34.0 (32.0, 36.1)	0.32	46.0 (40.5, 51.5)	41.7 (40.5, 42.8)	0.12	21.5 (12.2, 30.7)	30.9 (28.4, 33.5)	0.07	44.3 (1.6, 86.9)	40.1 (37.4, 42.8)	0.85
Both purposes	12.6 (9.3, 15.9)	17.8 (17.1, 18.5)	0.008	15.1 (1.6, 28.6)	17.6 (15.9, 19.3)	0.73	11.3 (7.9, 14.8)	17.4 (16.6, 18.3)	0.004	19.9 (8.4, 31.3)	18.6 (16.5,20.7)	0.82	18.9 (0.01, 46.5)	19.3 (17.0, 21.6)	0.98

*P*-values are from t-tests of the breast or prostate cancer variable coefficient in the models, not a comparison between predicted marginals. Covariates: leisure time physical activity level, age, weight status, education, marital status, employment status, functional status, difficulty walking, health insurance status, and survey year

### Walking volume

For women who walked (*n* = 4606), there were no significant differences between cancer survivors and those without cancer for median minutes of weekly walking, except for leisure walking (Table 4). For leisure walking, women cancer survivors reported 120 min (95% CI: 120, 140), and women without cancer reported 100 min (95% CI: 100, 105) (*p* = 0.0002). There were no significant racial/ethnic differences in median minutes of walking between cancer survivors and those without cancer, except for leisure walking among non-Hispanic white women: 120 min (95% CI: 120, 140) and 105 min (95% CI: 105, 120), respectively (*p* = 0.002).

**Table 4:** Weekly walking behavior volume (minutes) for cancer survivors and those without cancer, NHIS 2015 and 2020

	Cancer survivors *n* = 3667			Those without cancer *n* = 33,169			*p*
	Median 95% CI	Q1	Q3	Median 95% CI	Q1	Q3	
Overall							
Total	210 (180, 240)	105	390	180 (180, 195)	100	350	0.18
Transportation	60 (60, 75)	30	120	60 (60, 70)	30	140	0.04
Leisure	120 (120, 140)	60	225	100 (100,105)	60	210	< 0.0001
Women							
Total	210 (180, 245)	110	360	180 (180, 200)	105	355	0.41
Transportation	60 (60, 75)	30	120	60 (60, 70)	30	140	0.68
Leisure	120 (120,140)	60	225	100 (100, 105)	60	210	0.0002
Men							
Total	210 (165, 270)	90	420	180 (180, 200)	100	345	0.32
Transportation	60 (60, 90)	30	120	60 (60, 70)	30	140	0.01
Leisure	120 (120, 140)	60	225	100 (100, 105)	50	210	0.001

*P*-values are from Mood’s design-based median test comparing cancer survivors and those without cancer

For men who walked (*n* = 4485), there was not a significant difference in the median minutes of any walking (Table 4). For transportation walking, men cancer survivors reported 60 min (95% CI: 60, 90) and men without cancer reported 60 min (95% CI: 60, 70) (*p* = 0.01). For leisure walking, cancer survivors reported 120 min (95% CI: 120, 140) and those without cancer reported 100 min (95% CI: 100, 105) (*p* = 0.001). There were no significant racial/ethnic differences in median minutes of walking between cancer survivors and those without cancer, except for leisure walking among non-Hispanic white men: 120 min (95% CI: 120, 140) and 100 min (95% CI: 100, 105), respectively (*p* = 0.001).

## Discussion

This study estimated walking prevalence and volume overall and by walking purpose for cancer survivors and those without cancer stratified by sex and race/ethnicity. Walking is a light to moderate intensity PA amenable to cancer survivors starting with treatment through re-entry and into survivorship [27]. Estimating walking behaviors (prevalence and volume) provides understanding of an important component of overall PA and yet has not been previously reported. As walking is the most common type of physical activity, examining different purposes of walking may help provide insight into the motivation, purposes, and context of each walking behavior and the participants who engage in each type of walking. These factors are important to understand, because it may help providers and policymakers to support walking for cancer survivors as a key form of physical activity.

This analysis showed that there are no significant differences in walking prevalence for women, but men cancer survivors reported less overall walking, walking for transportation, or walking for both purposes. Volume of leisure walking was higher for cancer survivors compared to those without cancer. Some of these findings may have been expected, as it agrees with existing literature that finds similar MVPA levels between cancer survivors and those without cancer [5, 32], even though walking is generally light intensity PA. The analysis of walking prevalence by cancer site showed that walking was not different by race/ethnicity for breast cancer survivors, which agrees with previous research finding general PA levels comparable for Hispanic and non-Hispanic white breast cancer survivors and population controls [48]. Walking for both purposes was lower for men prostate cancer survivors overall and non-Hispanic white prostate cancer survivors compared to those without cancer, whereas a study not stratified by race and ethnicity found prostate cancer survivors reported more leisure time MVPA compared to those without cancer [33].

Results for the comparison of minutes of walking showed that cancer survivors reported more minutes of leisure walking compared to those without cancer. Also, non-Hispanic white women and men cancer survivors reported more minutes of leisure walking compared to those without cancer. These results on volume of walking also may have been expected as it aligns with research findings of cancer survivors reporting higher levels of leisure-time PA compared to the general population [7, 33]. While we did not specifically examine differences in walking for cancer survivors between racial and ethnic groups, these comparisons are possible with the presented point estimates and confidence intervals. For example, among cancer survivors, the prevalence of any walking for non-Hispanic white women was 69.3% (95% CI: 67.0%, 71.6%), and for non-Hispanic Black women was 59.9% (95% CI: 57.5%, 62.2%), suggesting a difference that has not consistently been found when examining only leisure-time PA [4, 9, 10, 49, 50].

Overall, the results from this study may have occurred because about 80% of women and 76% of men who report any weekly walking also report meeting PA guidelines [51]. Therefore, the general agreement in PA levels between cancer survivors and those without cancer [5, 32] is also reflected in the general agreement found in this study for walking prevalence; however, cancer survivors did report higher leisure walking volume. Previous literature has used meeting PA guidelines (yes/no) as the comparison measure between cancer survivors and those without cancer [5, 7, 10] and another only examined middle-aged adults [32]. Assessing walking behaviors by purpose and minutes adds more nuance to the existing literature and this study’s overall findings. For example, key walking behavior differences appeared for non-Hispanic white women and men. We do not have a ready explanation for these differences, but it does correspond with previous research that found significant PA associations with quality-of-life outcomes are different by race/ethnicity and by PA category (occupational or leisure) [48].

Implications of disaggregating walking by purpose for understanding walking behaviors of cancer survivors are that it may capture important contextual, social, or environmental conditions that aggregate measures of leisure-time PA may not capture. A finding of this study that men cancer survivors reported less walking for transportation or for both leisure and transportation is novel. While this was not found for women, potential explanations may be due to differences in car ownership, neighborhood amenities, or personal preferences between men cancer survivors and men without cancer. Future studies on the physical activity behaviors of cancer survivors may consider more PA domains (such as for transportation) and motivations or environmental supports for why survivors engage in certain PA domains, in addition to the common measure of aggregate leisure-time PA.

An important consideration when assessing PA and walking in the cancer survivor population is physical function and ability and perceived health (i.e., self-rated health), which generally decrease with increasing age [4, 52]. This research explored this facet (that the cancer survivor population was older than those without cancer) by examining survey questions about physical functional limitations and difficulty walking between cancer survivors and the general population, finding a higher prevalence of any functional limitations in the cancer survivor group, 33.9%, compared to those without cancer, 17.2%. Similarly, any reported difficulty walking in the cancer survivor group was 10.7%, compared to those without cancer which was 3.8%. From these two questions, it appears that cancer survivors overall may have increased physical barriers to PA and walking specifically; however, we did not find this consistently in our results. Walking prevalence overall and by race/ethnicity groups was generally not different between the two populations, even though the cancer survivor population was older on average and reported a higher prevalence of physical functional limitations and difficulty walking.

Another explanation of differences in walking behaviors may be cancer site-specific differences in prevalence, as explored by our analyses of breast and prostate cancer survivors. We did not detect differences in walking for breast cancer survivors and those without cancer. We did detect a difference in the prevalence of walking for both purposes, which was significantly lower in the prostate cancer survivor group, 12.6% (95% CI: 9.3%, 15.9%), compared to those without cancer, 17.8% (95% CI: 17.1%, 18.5%) (*p* = 0.008). This suggests a larger difference, when compared to the overall men cancer survivor group, which reported a prevalence of walking for both purposes: 14.7% (95% CI: 12.6%, 16.9%) compared to those without cancer 17.6% (95% CI: 16.9%, 18.3%) (*p* = 0.02). Although we did not statistically test the comparison between prostate cancer survivors and all men cancer survivors, it suggests cancer site may play a role in the ability or difficulty in participating in PA and walking [52, 53]. In addition to the benefits of walking for cancer survivors, who are older on average than those without cancer, there are other benefits of walking for chronic disease prevention especially salient to older populations, such as reduced risk of cardiovascular disease and type 2 diabetes management [54, 55].

Although PA is generally recommended for cancer survivors, as it is for those without cancer [13, 15, 56], many barriers to PA and walking need to be overcome to increase prevalence and minutes overall and between racial and ethnic groups, such as perceptions of safety of the environment [35, 36, 57], neighborhood disadvantage or multiple levels of factors [11, 49], and affordance of the environment for walking [58–60]. Creating safe, inclusive, diverse, and accessible neighborhoods are demonstrated factors associated with increased PA and walking [61].

### Limitations

Using self-reported behaviors and health on surveys has some limitations. First, survey respondents may be healthier and, in general, have lower mortality rates compared to the general population, which includes adults living in institutions or are otherwise under-represented in surveys, such as the unhoused [62]. Second, the 2015 NHIS was conducted in-person, whereas the 2020 NHIS was conducted mostly by phone due to COVID-19, resulting in declining response rates [63]. This may have had an effect on the representativeness of the survey that may not be adequately addressed with survey weighting and variance estimation.

Estimates of PA (and walking) may vary depending on the survey analyzed, how the questions are phrased (and changes between years), inclusion of relevant variables related to walking for transportation or leisure (e.g., car ownership), or measurement type (i.e., self-reported or device measured) [64]. Additionally, there is some uncertainty about using the leisure-time PA question to control for overall PA and whether there is overlap with the walking behavior questions. First, the leisure-time PA question likely does not capture other domains of PA such as transportation or occupation and is likely an underestimate of overall PA. Second, it is unclear the extent of overlap with reported walking behaviors. The leisure-time PA question only asks about moderate and vigorous activity, not light intensity activity. Some respondents may not consider their leisure-time walking to be of moderate or vigorous intensity, so they may not consider it in their response. Most likely, walking for transportation would not be included in a response to this question, so there is not likely overlap for this walking purpose. Previous examinations compared NHIS walking prevalence estimates, including and excluding adjustment by leisure-time PA, and found that the point estimates were slightly perturbed, but statistical significance remained [34].

Some other limitations to these analyses were due to an incomplete set of survey questions regarding cancer history. Neither stage at diagnosis nor treatment type were included. Both factors could have implications on walking behaviors and may explain some of the differences in prevalence between racial/ethnic groups. For example, non-Hispanic Black men have later stage at diagnosis for prostate cancer compared to non-Hispanic white men [65]. This may result in different disease management and treatment decisions, which may affect PA levels [66], with evidence suggesting that more severe treatment side effects, due in part to treatment type, are related to lower levels of PA in prostate cancer survivors [67].

It was difficult to fully account for the role of age in these analyses. While cancer survivors tend to be older on average than those without cancer, we would expect that health behaviors in general would be different from the younger population of those without cancer. While controlling for age in the adjusted predicted prevalence may account for some of the variation in health behaviors, it would not completely account for differences due to age. While stratification by age may yield better comparisons between these two groups, the small sample sizes for cancer survivors in age groups less than 65 would result in imprecise point estimates and large confidence intervals. Finally, age may be a confounder in the associations between walking, race and ethnicity, physical functional and difficulty walking status, and cancer survivor status [53]. While we controlled for age, it might not completely address how age interacts with these associations. Further stratification of cancer survivors and those without cancer, in addition to sex and race/ethnicity by age, was not statistically reliable due to the very small number of observations in the subgroups. Future research may use larger samples or novel analysis methods to address age when examining differences for racial and ethnic groups in the cancer survivor and general population for PA and walking behaviors.

## Conclusion

Walking is an important component of aerobic physical activity and is beneficial for cancer survivors’ treatment, recovery, and prevention of recurrence. This study did not find significant differences in walking prevalence for women, but men cancer survivors reported less overall walking, walking for transportation, or walking for both purposes. Volume of leisure walking is higher overall for cancer survivors compared to those without cancer. This suggests that despite potential health or functional differences between cancer survivors and those without cancer, leisure walking is an accessible and important source of physical activity.

## Supplementary information

Below is the link to the electronic supplementary material.

Supplementary file1 (DOCX 40 KB)

## Data Availability

No datasets were generated or analysed during the current study.
