# Supplementary material for: Estimates of walking prevalence and volume for U.S. cancer survivors and those without cancer: overall, by sex, and by race and ethnicity
Source: J Cancer Surviv. 2024 Dec 19;20(1):347–59. doi: 10.1007/s11764-024-01729-6 (PMC12906567; doi:10.1007/s11764-024-01729-6)
Supplement: Supplementary file 1 — Supplementary file1 (DOCX 40 KB) [file 11764_2024_1729_MOESM1_ESM.docx]

Supplement for: “Racial and Ethnic Differences in Walking Prevalence Between U.S. Cancer Survivors and Those Without Cancer”

List of Tables

[Table S1. Cancer type in analytic sample by survey year NHIS 2015 and 2020. 2](#_Toc156381215)

[Table S2. Unadjusted weighted walking prevalence (% and 95% CI) comparing cancer survivors to those without cancer for women overall and stratified by race/ethnicity, NHIS 2015 and 2020. 3](#_Toc156381216)

[Table S3. Unadjusted weighted walking prevalence (% and 95% CI) comparing cancer survivors to those without cancer for men overall and stratified by race/ethnicity, NHIS 2015 and 2020. 3](#_Toc156381217)

[Table S4. Unadjusted weighted walking prevalence (% and 95% CI) comparing female breast cancer survivors to those without cancer overall and stratified by race/ethnicity, NHIS 2015 and 2020. 4](#_Toc156381218)

[Table S5. Unadjusted weighted walking prevalence (% and 95% CI) comparing male prostate cancer survivors to those without cancer overall and stratified by race/ethnicity, NHIS 2015 and 2020. 4](#_Toc156381219)

# Table S1. Cancer type in analytic sample by survey year NHIS 2015 and 2020.

|  | 2015 | 2020 | Total |
| --- | --- | --- | --- |
| Reported Cancer Types | n | n |  |
| Bladder | 70 | 69 | 139 |
| Blood | 11 | 13 | 24 |
| Bone | 17 | 13 | 30 |
| Brain | 18 | 21 | 39 |
| Breast | 518 | 537 | 1055 |
| Cervix | 128 | 117 | 245 |
| Colorectal | 188 | 144 | 332 |
| Esophagus | 8 | 10 | 18 |
| Gallbladder | 1 | 2 | 3 |
| Head and neck | - | 30 | 30 |
| Kidney | 62 | - | 62 |
| Larynx | 7 | 3 | 10 |
| Leukemia | 33 | 39 | 72 |
| Liver | 22 | 15 | 37 |
| Lung | 79 | 72 | 151 |
| Lymphoma | 86 | 84 | 170 |
| Melanoma | 203 | 186 | 389 |
| Mouth/tongue/lip | 9 | 10 | 19 |
| Ovary | 65 | 56 | 121 |
| Pancreas | 19 | 11 | 30 |
| Prostate | 344 | 393 | 737 |
| Skin (non-melanoma) | 559 | 757 | 1316 |
| Skin (unknown kind) | 202 | 177 | 379 |
| Soft tissue | 10 | - | 10 |
| Stomach | 25 | 14 | 39 |
| Testis | 14 | - | 14 |
| Throat | 26 | 17 | 43 |
| Thyroid | 69 | 87 | 156 |
| Uterus | 109 | 79 | 188 |
| Other | 123 | 213 | 336 |

- not asked in survey year

# Table S2. Unadjusted weighted walking prevalence (% and 95% CI) comparing cancer survivors to those without cancer for women overall and stratified by race/ethnicity, NHIS 2015 and 2020.

|  | | **Women Overall** | | | **Women by Race/ethnicity** | | | | | | | | | | | |  |
| --- | --- | --- | --- | --- | --- | --- | --- | --- | --- | --- | --- | --- | --- | --- | --- | --- | --- |
|  |  |  |  |  | **Hispanic** | | | **White, non-Hispanic** | | **Black, non-Hispanic** | | | | **Other, non-Hispanic** | | | |
|  | n | Cancer Survivors  n=3,166 | Those without cancer n=25,814 | p | Cancer Survivors  n=197 | Those without cancer n=4,133 | p | Cancer Survivors  n=2,648 | Those without cancer n=16,080 | p | Cancer Survivors  n=193 | Those without cancer n=3,366 | p | Cancer Survivors  n=128 | Those without cancer n=2,235 | p | |
| Any reported walking | 19,788 | 65.0  (62.7, 67.2) | 68.5  (67.7, 69.3) | 0.003 | 68.3  (58.9, 76.8) | 66.0  (63.9, 68.2) | 0.62 | 65.4  (63.0, 67.8) | 70.3  (69.3, 71.2) | 0.0003 | 50.3  (40.9, 59.7) | 60.1  (57.7, 62.5) | 0.04 | 70.1  (59.2, 76.9) | 73.3  (70.9, 75.6) | 0.52 | |
| Transportation only | 2,155 | 5.4  (4.2, 6.7) | 7.4  (6.9, 7.8) | 0.002 | 14.0  (6.4, 25.4) | 9.4  (8.3, 10.7) | 0.33 | 4.6  (3.5, 5.9) | 5.8  (5.3, 6.4) | 0.05 | 7.9  (4.0, 13.6) | 10.6  (9.2, 12.2) | 0.26 | 3.6  (0.9, 9.3) | 9.4  (8.0, 11.0) | 0.006 | |
| Leisure only | 12,992 | 47.3  (44.9, 49.8) | 45.8  (44.9, 46.7) | 0.25 | 44.5  (33.9, 55.4) | 41.7  (39.5, 43.9) | 0.61 | 48.3  (45.7, 50.9) | 49.4  (48.2, 50.5) | 0.45 | 34.3  (25.3, 44.2) | 35.0  (32.7, 37.3) | 0.88 | 50.0  (38.5, 61.6) | 44.7  (41.9, 47.6) | 0.36 | |
| Both purposes | 4,641 | 12.3  (10.8, 13.9) | 15.3  (14.7, 16.0) | 0.0003 | 9.9  (5.7, 15.7) | 14.9  (13.5, 16.3) | 0.06 | 12.6  (10.8, 14.5) | 15.1  (14.2, 15.9) | 0.01 | 8.2  (4.3, 13.7) | 14.5  (12.8, 16.4) | 0.01 | 16.5  (8.9, 26.9) | 19.2  (16.9, 21.6) | 0.54 | |

p-values from adjusted Wald tests; n are unweighted

# Table S3. Unadjusted weighted walking prevalence (% and 95% CI) comparing cancer survivors to those without cancer for men overall and stratified by race/ethnicity, NHIS 2015 and 2020.

|  | | **Men Overall** | | | **Men by Race/ethnicity** | | | | | | | | | | | |
| --- | --- | --- | --- | --- | --- | --- | --- | --- | --- | --- | --- | --- | --- | --- | --- | --- |
|  |  |  |  |  | **Hispanic** | | | **White, non-Hispanic** | | **Black, non-Hispanic** | | | | **Other, non-Hispanic** | | |
|  | n | Cancer Survivors  n=2,504 | Those without cancer n=23,058 | p | Cancer Survivors  n=113 | Those without cancer n=3,573 | p | Cancer Survivors  n=2,180 | Those without cancer n=15,086 | p | Cancer Survivors  n=153 | Those without cancer n=2,325 | p | Cancer Survivors  n=58 | Those without cancer n=2,074 | p |
| Any reported walking | 17,048 | 62.5  (59.9, 65.0) | 66.0  (65.1, 66.9) | 0.01 | 63.6  (51.2, 74.8) | 60.5  (58.3, 62.6) | 0.59 | 62.8  (60.1, 65.5) | 67.9  (66.8, 68.9) | 0.0006 | 50.1  (40.0, 60.2) | 60.9  (58.3, 63.4) | 0.04 | 78.3  (60.6, 90.7) | 69.6  (66.9, 72.2) | 0.24 |
| Transportation only | 2,409 | 5.9  (4.8, 7.1) | 9.2  (8.6, 9.7) | <.0001 | 6.6  (2.0, 15.3) | 8.8  (7.7, 10.0) | 0.46 | 5.7  (4.5, 7.1) | 8.7  (8.1, 9.4) | <.0001 | 8.3  (4.7, 13.2) | 11.4  (9.6, 13.3) | 0.16 | 5.7  (1.4, 14.5) | 10.2  (8.7, 11.9) | 0.16 |
| Leisure only | 10,123 | 44.1  (41.6, 46.6) | 39.0  (38.1, 39.9) | 0.0001 | 43.0  (31.1, 55.6) | 34.0  (32.0, 36.1) | 0.14 | 45.0  (42.4, 47.6) | 41.6  (40.4, 42.7) | 0.02 | 27.7  (19.4, 37.4) | 30.8  (28.2, 33.5) | 0.51 | 51.0  (33.1, 68.6) | 40.1  (37.3, 43.0) | 0.23 |
| Both purposes | 4,516 | 12.6  (10.8, 14.5) | 17.8  (17.1, 18.6) | <.0001 | 14.0  (7.5, 23.2) | 17.6  (15.9, 19.4) | 0.36 | 12.1  (10.3, 14.2) | 17.5  (16.7, 18.4) | <.0001 | 14.0  (7.7, 22.8) | 18.7  (16.5, 21.1) | 0.21 | 21.6  (9.5, 38.9) | 19.3  (17.0, 21.8) | 0.74 |

p-values from adjusted Wald tests; n are unweighted

# Table S4. Unadjusted weighted walking prevalence (% and 95% CI) comparing female breast cancer survivors to those without cancer overall and stratified by race/ethnicity, NHIS 2015 and 2020.

|  | **Women Overall** | | | **Women by Race/ethnicity** | | | | | | | | | | | | |
| --- | --- | --- | --- | --- | --- | --- | --- | --- | --- | --- | --- | --- | --- | --- | --- | --- |
|  |  |  |  | **Hispanic** | | | **White, non-Hispanic** | | | **Black, non-Hispanic** | | | | **Other, non-Hispanic** | | |
|  | Breast Cancer Survivors | Those without cancer | p | Breast Cancer Survivors | Those without cancer | p | Breast Cancer Survivors | Those without cancer | p | | Breast Cancer Survivors | Those without cancer | p | Breast Cancer Survivors | Those without cancer | p |
| Any reported walking | 64.5  (60.2, 68.6) | 68.5  (67.7, 69.3) | 0.06 | 64.5  (47.2, 79.3) | 66.0  (63.9, 68.2) | 0.84 | 65.3  (60.3, 70.0) | 70.3  (69.3, 71.2) | 0.05 | | 50.9  (36.5, 65.3) | 60.1  (57.7, 62.5) | 0.20 | 70.8  (52.8, 85.1) | 73.3  (70.9, 75.6) | 0.75 |
| Transportation only | 7.3  (4.7, 10.7) | 7.4  (6.9, 7.8) | 0.96 | 26.0  (9.01, 50.7) | 9.42  (8.26, 10.7) | 0.16 | 6.0  (3.7, 9.2) | 5.8  (5.3, 6.4) | 0.87 | | 3.5  (0.5, 11.3) | 10.6  (9.2, 12.2) | 0.007 | 2.9  (0.2, 12.2) | 9.4  (8.0, 11.0) | 0.03 |
| Leisure only | 44.7  (40.3, 49.1) | 45.8  (44.9, 46.7) | 0.60 | 28.0  (15.6, 43.3) | 41.7  (39.5, 43.9) | 0.08 | 46.5  (41.6, 51.5) | 49.4  (48.2, 50.5) | 0.25 | | 36.9  (23.5, 52.0) | 35.0  (32.7, 37.3) | 0.78 | 52.7  (33.0, 71.8) | 44.7  (41.9, 47.6) | 0.24 |
| Both purposes | 12.5  (9.7, 15.8) | 15.3  (14.7, 16.0) | 0.07 | 10.5  (4.7, 19.5) | 14.9  (13.5, 16.3) | 0.26 | 12.7  (9.5, 16.6) | 15.1  (14.2, 15.9) | 0.19 | | 10.5  (4.2, 20.8) | 14.5  (12.8, 16.4) | 0.31 | 15.3  (5.14, 32.2) | 19.2  (16.9, 21.6) | 0.55 |

p-values from adjusted Wald tests

# Table S5. Unadjusted weighted walking prevalence (% and 95% CI) comparing male prostate cancer survivors to those without cancer overall and stratified by race/ethnicity, NHIS 2015 and 2020.

|  | **Men Overall** | | | **Men by Race/ethnicity** | | | | | | | | | | | | |
| --- | --- | --- | --- | --- | --- | --- | --- | --- | --- | --- | --- | --- | --- | --- | --- | --- |
|  |  |  |  | **Hispanic** | | | **White, non-Hispanic** | | | **Black, non-Hispanic** | | | | **Other, non-Hispanic** | | |
|  | Prostate Cancer Survivors | Those without cancer | p | Prostate Cancer Survivors | Those without cancer | p | Prostate Cancer Survivors | Those without cancer | p | | Prostate Cancer Survivors | Those without cancer | p | Prostate Cancer Survivors | Those without cancer | p |
| Any reported walking | 61.3  (56.6, 65.9) | 66.0  (65.1, 66.9) | 0.05 | 69.1  (50.5, 84.0) | 60.5  (58.3, 62.6) | 0.31 | 62.8  (57.6, 67.8) | 67.9  (66.8, 68.9) | 0.05 | | 48.6  (35.6, 61.7) | 60.9  (58.3, 63.4) | 0.07 | 61.4  (25.4, 89.9) | 69.6  (66.9, 72.2) | 0.62 |
| Transportation only | 5.5  (3.5, 8.1) | 9.2  (8.6, 9.7) | 0.001 | 8.6  (1.4, 25.7) | 8.8  (7.7, 10.0) | 0.97 | 4.8  (2.6, 7.9) | 8.7  (8.1, 9.4) | 0.003 | | 7.8  (3.7, 14.2) | 11.4  (9.6, 13.3) | 0.18 | 5.6  (0.4, 22.1) | 10.2  (8.7, 11.9) | 0.38 |
| Leisure only | 45.4  (40.7, 50.2) | 39.0  (38.1, 39.9) | 0.008 | 45.3  (26.4, 65.3) | 34.0  (32.0, 36.1) | 0.25 | 49.2  (43.8, 54.5) | 41.6  (40.4, 42.7) | 0.006 | | 24.6  (15.3, 36.0) | 30.8  (28.2, 33.5) | 0.25 | 39.9  (9.6, 77.6) | 40.1  (37.3, 43.0) | 0.99 |
| Both purposes | 10.4  (7.9, 13.5) | 17.8  (17.1, 18.6) | <.0001 | 15.2  (4.6, 33.4) | 17.6  (15.9, 19.4) | 0.72 | 8.9  (6.4, 12.0) | 17.5  (16.7, 18.4) | <.0001 | | 16.2  (7.4, 29.0) | 18.7  (16.5, 21.1) | 0.62 | 16.0  (1.7, 48.8) | 19.3  (17.0, 21.8) | 0.77 |

p-values from adjusted Wald tests
